# i Sexual function in breast cancer patients: a prospective study from Iran

**DOI:** 10.1186/1756-9966-31-20

**Published:** 2012-03-09

**Authors:** Iraj Harirchi, Ali Montazeri, Fereshteh Zamani Bidokhti, Nina Mamishi, Kazem Zendehdel

**Affiliations:** 1 Cancer Research Centre, Cancer Institute of Iran, Tehran University of Medical Sciences, Tehran, Iran; 2 Mental Health Research Group, Health Metrics Research Centre, Iranian Institute for Health Sciences Research, ACECR, Tehran, Iran; 3 Department of Medical Epidemiology and Biostatistics, Karolinska Institute, Stockholm, Sweden

## Abstract

**Background:**

Sexual function in patients with breast cancer especially in younger patients is an important issue from clinical and psychosocial perspectives. This study aimed to assess sexual function among Iranian breast cancer patients.

**Methods:**

This was a prospective study of sexual function in breast cancer patients attending the Cancer Institute of Iran. Sexual function was assessed using the Female Sexual Function Index (FSFI) at two points in time: baseline (pre-treatment) and after completion of cancer treatment at follow-up visits (post-treatment). Pre- and post-treatment data were compared. In addition logistic regression analysis was performed to find out factors that contributing to post-treatment sexual dysfunction.

**Results:**

In all 277 breast cancer patients were approached. Of these, 231 patients (83%) were sexually active and data for 216 patients (93.5% of sexually active patients) were available at pre-and post-treatment. Overall pre- and post-treatment sexual dysfunction was found to be 52% and 84%, respectively indicating a significant deterioration in sexual function among breast cancer patients. The results obtained from multiple logistic regression analysis indicated that younger age [OR = 0.95, 95% CI = 0.93-0.98; *P *= 0.04], receiving endocrine therapy [OR = 3.34, 95% CI = 1.37-7.91; *P *= 0.007] and poor sexual function at pre-treatment [OR = 12.3, 95% CI = 3.93-39.0; *P *< 0.0001] were the most significant contributing factors to post-treatment sexual disorders.

**Conclusion:**

A significant number of breast cancer patients experience deterioration in sexual function over time. The findings from this study indicated that younger age, receiving endocrine therapy, and poor sexual function at diagnosis were the most significant predicting factors for sexual disorders following treatment.

## Background

Studying sexual function in women who lose their breasts due to breast cancer and are sexually active is vital issue from both clinical and psychosocial perspectives [1]. A study on sexual quality of life in women with newly diagnosed breast cancer indicated that **a**bout 60% of breast cancer patients reported disruption in their sexual quality of life [2]. A recent publication on sexual function after breast cancer studying 1,011 women reported that 70% of patients experienced sexual function problems. The study also indicated that several factors contributed to sexual function problems. Those who received aromatase inhibitors were more likely to experience more sexual function problems compared to those who received tamoxifien but in both group body image was the most contributing factor to sexual dysfunction [3]. These findings suggest that the impact of breast cancer on sexuality is much more complex than women simply losing their breasts or receiving different treatment modalities.

Studies have shown that disrupted sexual functioning or unsatisfactory sexual life was related to poorer quality of life at younger age, treatment with chemotherapy, total mastectomy, emotional distress consequent on an unsatisfactory sexual life, and difficulties with partners because of sexual relationships [4-8]. This latter factor was further examined and recently a French study found that 'no sexual activity' or 'sexual dissatisfaction' among breast cancer patients were associated with the feeling of emotional separation in the couple or of partner's fear of sexual intercourse [9]. Emilee et al. [10] in a review of sexuality after breast cancer highlighted the issue of 'women's intrapsychic' experience of changes to sexuality. They argued this experience includes a fear of loss of fertility, negative body image, feelings of sexual unattractiveness, loss of femininity, depression and anxiety, as well as alterations to a sense of sexual self. Then they concluded that sexuality in the context of breast cancer could not be conceptualized the physical body separately from women's intrapsychic experience.

With any interpretations sexual functioning seems important area that needs more attention, especially for younger breast cancer survivors. It is argued that younger survivors may need interventions that specifically target their needs related to menopausal symptoms and problems with relationships, sexual functioning and body image [11]. There is evidence that the quality of sexual life in breast cancer survivors could be improved with the sexual life reframing program focusing on the physical, psychological, and relational aspects of sexual health elements at couples rather than survivors only and if delivered earlier and for a longer period [12].

No study so far has reported on prevalence of sexual function among Iranian breast cancer patients. Breast cancer patients in Iran are usually younger that their western counterparts [13] and thus might report different experiences. In addition women in Islamic countries such as Iran usually have some reservations in talking about and reporting sexual problems or seeking processional help [14]. The aim of this study was to elucidate the issue and contribute to existing knowledge on the topic and provide necessary information for implementing possible future interventions in order to improve quality of life in breast cancer patients.

## Methods

### Patients and data collection

This was a prospective study of sexual function among breast cancer patients attending the Cancer Institute in Tehran, Iran. Patients were included in the study if they had confirmed diagnosis of breast cancer (any stages), were married and sexually active. Patients were assessed at two points in time: once before surgery and once after surgery and completion of adjuvant treatment (usually 3 months after chemotherapy or radiotherapy at first follow-up visits). Demographic and clinical data were collected at baseline and a sexual functioning questionnaire was completed for each patient at pre-and post-treatment assessments.

### Sexual function

Sexual function was assessed using the Female Sexual Function Index (FSFI). The FSFI is a 19-itmes questionnaire that contains six subscales: sexual desire, arousal, lubrication, orgasm, satisfaction and pain. It provides a score for each subscale as well as a total score for the whole questionnaire. The total score ranges from 2 to 36 with higher scores indicating a better sexual function [15]. We used the Iranian version of the questionnaire. The psychometric properties of the Iranian version are well documented. The cut-off point for sexual disorder for Iranian females was found to be 28 [16].

### Statistical analysis

The analysis was restricted to patients for whom both pre-and post-treatment data were available. In addition to descriptive statistics, paired sample t-test was used to compare sexual function before and after treatment. Relative to cut-off point on the FSFI (less than 28 versus 28 or above), patients with and without sexual disorders at post-treatment were indicated and the contribution of demographic and clinical factors to sexual disorder was investigated by performing both univariate and multiple logistic regression analyses.

### Ethics

The ethics committee of Tehran University of Medical Sciences approved the study. All patients gave their written informed consent.

## Results

In all 277 patients with breast cancer were approached. Of these 231 patients (83%) were sexually active and were included in the study. Since 15 patients did not complete the questionnaire at follow-up due to dislike, the data for 216 patients (93.5% of sexually active patients) were available for both pre-and post-treatment evaluations. There were no significant score differences on the FSFI between those who did not participate at follow-up assessment and the rest of patients (n = 216) at baseline (the results are not show and is available from the corresponding authors). The characteristics of patients and the mean duration follow-up (time interval between pre- and post-treatment evaluations) are presented in Table 1.

**Table 1:** The characteristics of the study sample (n = 216)

		**No**.	%
**Demographic status**			

*Age*			

	≤ 40		

	41-45	45	20.8

	46-50	51	23.6

	51-55	47	21.8

	56 ≥	32	14.8

	Mean (SD)	44.3 (8.6)	

*Education*			

	Illiterate		

	Primary	111	51.4

	Secondary	61	28.2

	Higher	16	7.4

*Employment*			

	Housewife		85.6

	Employed	31	14.4

**Clinical status**			

*Disease stage*			

	I		

	II	91	42.1

	III	39	18.1

	Unknown	54	25.0

*Surgery*			

	Conservative		

	Mastectomy	156	72.2

*Chemotherapy*			

	Yes	200	92.6

	No	16	7.4

*Radiotherapy*			

	Yes	187	86.6

	No	29	13.4

*Endocrine therapy*			

	Yes	162	75.0

	No	54	25.0

**Sexual status**			

*Age at marriage*			

	Mean (SD)	19.1 (4.2)	-

*Age at first intercourse*			

	Mean (SD)	19.3 (4.2)	

*Intercourse per week*			

	1-2 times	196	90.7

	3-4 times	17	7.9

	> 4 times	3	1.4

**Time interval between pre- and post-treatment evaluations (months)**	Mean (SD)	9.1 (1.06)	

The mean score of patients on the FSFI at pre-and post-treatment was 26.6 (SD = 4.26) and 22.1 (SD = 5.89) respectively indicating a significant deterioration in sexual function among the study sample at post-treatment (*P *< 0.0001). At post-treatment assessment scores for sexual desire and lubrication showed greater decrease compared to other domains. The findings indicated that 52% of breast cancer patients at pre-treatment and 84% at post-treatment were suffering from poor sexual function. The results are shown in Table 2.

**Table 2:** Pre- and post-treatment sexual functioning in breast cancer patients as measured by the Female Sexual Function Index-FSFI (higher scores indicate a better function, n = 216)

	Pre-treatment	Post-treatment		
	**Mean (SD)**	**Mean (SD)**	**Effect size**	P*

**FSFI domains**				

Sexual desire	3.8 (0.97)	2.8 (1.13)	0.95	< 0.001

Arousal	4.1(1.25)	3.2 (1.45)	0.66	< 0.001

Lubrication	5.3(1.01)	4.3 (1.48)	0.79	< 0.001

Orgasm	4.8(1.17)	4.0 (1.47)	0.60	< 0.001

Satisfaction	3.3(1.47)	3.0 (1.26)	0.22	< 0.001

Pain	5.2(1.19)	4.5 (1.63)	0.49	< 0.001

**Total FSFI score**	26.6 (4.26)	22.1 (5.89)	0.87	< 0.001

Range	7.2-34.2	2.8-32.9	-	-

**Sexual disorder†**	Number (%)	Number (%)		< 0.0001¶

No	103 (48)	34 (16)		

Yes	113 (52)	182 (84)	-	-

* Derived from paired t-test.

**† **According to cut-off point score for Iranian females [16].

¶ Derived from Chi-square test.

The results obtained from multiple logistic regression analysis indicated that the most significant contributing factors to sexual disorder at post-treatment were younger age [OR = 0.95, 95% CI = 0.93-0.98; *P *= 0.04], receiving endocrine treatment [OR = 3.34, 95% CI = 1.38-8.06; *P *= 0.007], and poorer sexual dysfunction at pre-treatment [OR = 12.3, 95% CI = 3.93-39.0; *P *< 0.0001]. Other variables in the model did not show any significant results. Table 3 presents the findings.

**Table 3:** The results obtained from logistic regression indicating factors predicting sexual dysfunction at post treatment in breast cancer patients (n = 216)

	OR (95% CI)*	P	OR (95% CI)**	P
**Age**	0.96 (0.94-0.99)	0.05	0.95 (0.93-0.98)	0.04

**Education**				

Illiterate	1.0 (ref.)		1.0 (ref.)	

Primary	1.61 (0.56-4.61)	0.36	1.32 (0.36-4.80)	0.66

Secondary/higher	1.47 (0.49-4.40)	0.48	1.28 (0.32-5.01)	0.72

**Employment**				

Housewife	1.0 (ref.)		1.0 (ref.)	

Employed	1.03 (0.36-2.91)	0.94	1.55 (0.38-6.27)	0.53

**Surgery**				

Conservative	1.0 (ref.)		1.0 (ref.)	

Mastectomy	1.30 (0.55-3.05)	0.54	1.07 (0.36-3.22)	0.89

**Chemotherapy**				

No	1.0 (ref.		1.0 (ref.)	

Yes	1.88 (1.10-6.24)	0.03	1.34 (0.25-7.31)	0.73

**Radiotherapy**				

No	1.0 (ref.)		1.0 (ref.)	

Yes	1.88 (0.73-4.84)	0.18	2.30 (0.57-9.31)	0.24

**Endocrine therapy**				

No	1.0 (ref.)		1.0 (ref.)	

Yes	3.36 (1.57-7.22)	0.002	3.34 (1.38-8.06)	0.007

**Pre-treatment sexual dysfunction**				

No	1.0 (ref.)		1.0 (ref.)	

Yes	11.1 (3.78-33.1)	< 0.0001	12.3 (3.93-39.0)	< 0.0001

**Time interval between pre-and post-treatment evaluations (months)**	-	-	1.10 (0.33-3.63)	0.21

* Obtained from univariate logistic regression analysis

** Obtained from multiple logistic regression analysis (adjusted odds ratio)

## Discussion

The findings from this prospective study indicated that the prevalence of sexual dysfunction among Iranian breast cancer patients was relatively high. The findings also indicated that younger age, receiving endocrine therapy and pre-treatment sexual dysfunction were independent and significant contributing variables to post-treatment sexual disorders. It is well documented that endocrine effects of adjuvant therapy, especially chemotherapy, in younger survivors causes premature menopause that is associated with poorer quality of life, decreased sexual functioning, menopausal symptom distress, and psychosocial distress related to infertility [17], although it is believed that as a whole adjuvant endocrine therapy or radiation therapy for early stage breast cancer do not causes premature menopause. As noted by Cella and Fallowfield [18], recognition and management of treatment-related side-effects for breast cancer patients receiving adjuvant endocrine therapy is an important issue since such side-effects negatively affect sexual functioning, health-related quality of life and adherence to therapy. They argue that adverse events across all adjuvant endocrine trials regardless of the treatment, vasomotor symptoms such as hot flushes are the most common side effects. Other frequently reported side-effects such as vaginal discharge, vaginal dryness, dyspareunia, and arthralgia vary in prevalence between tamoxifen and aromatase inhibitors [18].

Although there were significant decreases in all measures at post-treatment assessment compared to pre-treatment evaluation, greater decrease was observed for sexual desire (3.8 vs. 2.8) and lubrication (5.3 vs. 4.3). Perhaps these are very important aspect of sexual life for women and should receive further attention when studying sexual issues in breast cancer patients. It has been shown that sexual desire and lubrication are two important affecting factors in breast cancer survivors after mastectomy [19]. In addition a study from Switzerland using the FSFI found that the only predictor for desire was quality of relationship while chemotherapy was predictive for problems with arousal, lubrication, orgasm, and sexual pain. The authors concluded that sexual dysfunction after breast cancer is common and thus women should be informed properly at an early stage of treatment. They suggested that specific interventions have to be offered considering person-related preexisting factors and couples at risk should be supported in the transition to a new sexual life after breast cancer [20].

In univariate analysis chemotherapy was found to have a significant association with post-treatment sexual disorder. However, in multiple logistic regression analysis this significant association was disappeared. One explanation for such observation might be due to the fact that we included endocrine therapy as an independent factor in the regression analysis and thus the hormonal side effects of endocrine therapy masked the hormonal side effects of chemotherapy in the final model. Although we adjusted the regression model for the time interval between pre-and post-treatment evaluations, another possibility for such results might be due to the fact that there were different time point for evaluations between the patients who received hormonal therapy and chemotherapy. In fact many patients received the chemotherapy and hormonal therapy together with sequential process.

Pretreatment sexual disorder appeared as important predicting factor for post-treatment sexual dysfunction. In fact many women indicated that they were suffering from sexual disorders even before diagnosis of breast cancer. This is why some investigators argued that the negative effects of cancer and its management on sexual function and satisfaction can be somewhat mitigated by understanding pre-diagnosis sexual functioning level [21]. A study indicated that two main issues affect breast cancer patients' sexuality after surgical treatment: personality and psychological factors. The study found that clinical factors did not predict quality of sexual life, sexual functioning and sexual enjoyment [22]. However, studies have shown that compared with pre-treatment levels considerably more women report moderate or severe problems with sexual interest and sexual activity over time. It was suggested that upper limb dysfunction, such as that caused by lymphedema, might be a significant factor that interfere with sexual functioning in breast cancer patients [23]. A recent publication reported that the presence of mood disorder, but not fatigue, demographic, or treatment variables, independently predicted worse overall sexual satisfaction. The study concluded that sexual dysfunction is common after breast cancer therapy and impacts quality of life and interventions should include identification and treatment of concomitant mood disorder [24]. A systematic review of the literature on interventions for sexual problems following treatment for breast cancer indicated that tentative findings of the studies under review suggest that the most effective interventions are couple-based psycho-educational interventions that include an element of sexual therapy [25]. However, as discussed by Krychman and Katz [26] sexual dysfunction during or following cancer therapy is a very complex disorder. They suggest that care and consultation between the survivor, her partner, the oncologists, and primary care practitioner should be aimed at discussing individualized treatment plans that minimize risk and maximize sexual wellness.

This study has some strengths including a prospective design, the use of a validated measure of sexual function and the fact that we are reporting from a diverse population where cultural and religious issues play important role in women's sexual life. For instance desire for sex by women (asking or showing interest in sex) is perceived negatively and always men must initiate; or the husband's preferences and satisfaction are more important than the wife's satisfaction and thus if husbands were satisfied, women tend to show that they are satisfied, too [27]. However, the present study suffers from limitations. We did not collect data on women's menopausal status or detailed data on the relative use of tamoxifien versus aromatase inhibitors by patients. This information might be necessary for regression analysis in order to have a better interpretation of the results.

## Conclusion

Breast cancer patients might show deterioration in sexual function over time. The findings from this study indicated that younger age, receiving endocrine therapy, and poor sexual function at diagnosis were the most significant predicting factors for sexual disorders in Iranian breast cancer patients following treatment.

## Competing interests

The authors declare that they have no competing interests.

## Authors' contributions

NM together with FZB contributed to the process of data collection, and data entry. IH contributed to design and patient recruitment. AM contributed to the analysis and wrote the paper. KZ contributed to design and analysis. All authors read and approved the final manuscript.
